# Risk factors of impaired fasting glucose and type 2 diabetes in Yaoundé, Cameroon: a cross sectional study

**DOI:** 10.1186/s12889-015-1413-2

**Published:** 2015-01-31

**Authors:** Clement Nyuyki Kufe, Kerstin Klipstein-Grobusch, Fezeu Leopold, Felix Assah, George Ngufor, George Mbeh, Vivian Nchanchou Mbanya, Jean Claude Mbanya

**Affiliations:** Division of Epidemiology & Biostatistics, School of Public Health, Faculty of Health Sciences, University of the Witwatersrand, Johannesburg, South Africa; Health of Populations in Transition (HoPiT) Research Group, Faculty of Medicine and Biomedical Sciences, The University of Yaoundé 1, Yaoundé, Cameroon; Julius Global Health, Julius Center for Health Sciences and Primary Care, University Medical Center Utrecht, Utrecht, The Netherlands; Université Paris 13, Sorbonne Paris Cité, Unité de Recherche en Epidémiologie Nutritionnelle (UREN)/U557 Inserm/U1125 Inra/Cnam/CRNH, Paris, France; Department of Community Medicine, University of Oslo, Oslo, Norway

**Keywords:** Diabetes, Impaired fasting glucose, Prevention, Non-communicable diseases, Low and middle income countries

## Abstract

**Background:**

The prevalence of diabetes is increasing worldwide, particularly in low and middle income countries, where treatment and control are often unavailable and inaccessible. Information on risk factors at local and regional levels is of utmost importance for tailored prevention programmes to curb the rise in diabetes.

The current study was undertaken to investigate the prevalence of Impaired Fasting Glucose (IFG)/Type 2 Diabetes (T2D) and its risk factors in the adult population in Biyem-Assi-Yaoundé, Cameroon.

**Methods:**

Information on cardiovascular risk factors using the WHO STEPwise approach was obtained for 1623 men and women aged 25 years and older of the CAMBoD Project in Biyem-Assi, Yaoundé, Cameroon. T2D was defined as fasting capillary glucose (FCG) ≥ 7.0 mmol/l and/or being on diabetes medication, IFG/T2D as FCG ≥ 6.1 mmol/l and/or being on diabetes medication. Descriptive statistics and multivariate logistic regression analyses were used to describe prevalence of IFG/T2D, prevalence of risk factors for IFG/T2D and to investigate the association of risk factors with prevalence of IFG/T2D.

**Results:**

Prevalence of T2D and of IFG/T2D was 3.3% and 5.7%. Prevalence of hypertension, obesity and abdominal obesity (elevated waist circumference) was 26.6%, 28.4% and 34.9%, respectively. Age and abdominal obesity were significantly associated with IFG/T2D in multivariate logistic regression.

**Conclusion:**

For successful primary prevention of T2D in the general population in Cameroon tailored efforts to address obesity, particularly abdominal obesity, and associated life-style factors are warranted.

## Background

Prevalence of type 2 diabetes (T2D) in both developed and developing countries is expected to increase dramatically from 246 million people (6.0%) aged 20 to 79 in 2007 [1] to 382 million in 2013 and to a projected 592 million people by 2035 [2]. About 175 million people with diabetes are undiagnosed. Diabetes caused 5.1 million deaths in 2013 and impact considerably on people under the age of 60 years. In Africa, 76.0% of deaths due to diabetes affected this age group [2]. The burden of T2D is particularly high in low and middle income countries [1-4]; for Africa prevalence of T2D was 4.5% in 2011, 5.7% in 2013 and is projected to increase to 6.0% by 2035 with the highest projected increase (109.6%) in the number of adults aged 20-79 years [2]. For Cameroon prevalence of T2D was 0.8% in 2003, projections for 2030 estimate diabetes prevalence to be 4.8% [5,6]. The rapid urbanisation in sub-Saharan Africa coupled with sedentary lifestyle and increased adiposity in urban areas is a major determinant in the increasing prevalence of diabetes and cardiovascular diseases. Urban residence is associated with a more than twice increased risk of diabetes or IFG [4]. IFG identifies risk state for future diabetes, cardiovascular disease and premature mortality. Determining IFG in asymptomatic individuals allows for prevention strategies to be implemented that can delay or prevent onset of T2D. Overall a prevalence of 5.0% and more for IFG has been observed, varying across different age groups and between populations [5]. Age-adjusted prevalence of IFG in World Health Organisation’s African region was 9.7% in 2011 as compared to global estimates of 6.5%. [5].

T2D is responsible for major contemporary causes of morbidity and mortality including cardiovascular diseases (CVD), retinopathies, nephropathies and neuropathies. Fifty percent of people living with T2D die of cardiovascular disease [2]. Most of T2D cases could be controlled and prevented via lifestyle modification. Factors that affect the onset of T2D are well known, such as age, ethnicity and modifiable risk factors like obesity, physical inactivity, dietary intake, tobacco and excessive alcohol consumption [6,7], though their prevalence and thus prevention potential may differ according to geographical and cultural context. IFG indicates above normal fasting blood glucose levels but lower than threshold for diagnosis of diabetes. IFG is a strong risk marker for the development of diabetes and associated with increased CVD risk [8]. Previous studies showed that during the five years preceding a diagnosis of T2D, 60.0% of people showed IFG or Impaired Glucose Tolerance (IGT) [9]. Screening for diabetes is not different from screening for IFG and the same risk factors associated with diabetes are associated with IFG [10]. Thus targeting individuals with IFG/T2D for prevention and control is of major importance to delay and/or prevent the onset of diabetes and complications which are a major cause of morbidity and mortality and highly related to duration, chronic levels of glycaemia and other risk factors.

The current study was undertaken to investigate the prevalence of modifiable risk factors and their association with IFG/T2D to guide primary prevention and control of IFG/T2D in an urban population in Cameroon.

## Methods

### Study population

A population-based survey to evaluate the burden of diabetes was conducted in Biyem-Assi, Yaoundé, Cameroon, in 2007 as part of the Cameroon Burden of Diabetes Project (CAMBoD), a multidisciplinary programme contributing to surveillance, prevention and control of diabetes in urban areas [11]. Biyem Assi health district had 9.7% of health facilities of the centre region, Republic of Cameroon, which comprised 25.1% of health facilities of the country in 2011 [12]. Biyem Assi, an urban area, with most inhabitants being government or private sector workers earning a salary, business men and women or students, has previously been shown to represent the urban population in Yaoundé [13]. Based on a census enlisting all adults aged 25 years and above living in the CAMoD study area in Biyem-Assi, Yaoundé participants were invited to participate in the 2007 survey. Multilevel systematic cluster sampling stratified by age group was used, a cluster being a household. The first household was selected randomly. Individual age group sampling was done by which every age group was represented thus avoiding having the sample size of the most populated age group within a limited spatial surface. Survey participation rate was 82.5% (n = 2062). Participants without the main dependent variable (FCG) were excluded from the analyses resulting in an analytic study sample of 1623 study participants.

### Data collection and management

Teams of four trained interviewers, each including a medical doctor, collected information on participants’ self-reported behavioural and lifestyle factors for chronic diseases, blood pressure, anthropometry measurements and blood samples for biochemical analysis from September to October 2007 using standardized procedures. For quality control purposes calibrated instruments were used and adherence of trained interviewers to standardised procedures was monitored.

Participants were asked to fast overnight. For measurement of fasting capillary glucose (FCG), study participants, who had not eaten or drank anything (except plain water) for at least eight hours were visited at home between 5:30 am and 9:00 am the next day. Assessment of IFG/T2D was based on fasting capillary glucose measurements with HemoCue®B-Glucose photometer, a validated instrument shown to give consistent readings as compared to other instruments [14]. The participant’s middle or ring finger, not bearing a ring, was cleaned with an alcohol swab and pricked at the side with a lancet. Blood was collected with a microcuvette, placed into a cuvette holder and results displayed immediately [15]. HemoCue®B-Glucose photometers were controlled every morning. Every sixth consecutive participant screened negative was administered an oral glucose tolerance test to ascertain reliability of results for FCG. IFG/T2D was defined as fasting capillary glucose (FCG) ≥6.1 mmol/l and/or on diabetes medication (insulin and/or anti-diabetic drugs) [8], participants with FCG between 6.1 mmol/l and 6.9 mmol/l were considered to have IFG, and those with FCG ≥7.0 mmol/l (126 mg/dl) and/or on diabetes medication (insulin and/or anti-diabetic drugs) were considered to have diabetes.

The World Health Organisation (WHO) STEPwise approach, a standardized questionnaire for chronic disease risk factor surveillance [16] including questions on diabetes and hypertension status (if diabetic or hypertensive and whether on diabetes or hypertensive medication (insulin and/or oral remedy)), smoking habits, alcohol intake, dietary intake and physical activity was applied by interviewers trained in the standardized administration of questionnaires in both English and French, the official languages in Cameroon. Participants previously on anti-diabetes medication and with FCG < 6.1 mmol/l and, participants informed by a health professional that s/he was diabetic but on local anti-diabetic remedy were excluded. The instruments for the survey were pretested and revised if indicated prior to the commencement of the survey.

Systolic (SBP) and diastolic (DBP) blood pressure was measured using fully automated calibrated Omron M3 machines with the participant being seated for at least five minutes, legs uncrossed and the left arm resting on a table prior to taking measurements. Blood pressure was measured thrice, the mean of the second and third measurement was considered for this study. Participants with mean SBP ≥ 140 and/or DBP ≥ 90 mmHg and/or on hypertensive medication were considered to be hypertensive [17].

Anthropometric measurements, including weight (to the nearest 0.1 kg, SECA scales), height (to the nearest 0.1 cm, locally made wood stadiometers), waist and hip circumferences (to the nearest 0.1 cm, flexible inelastic fibreglass meter band), were performed using standard methods. Waist circumference was measured over light clothing at the level of the midpoint between the inferior margin of the last rib and the crest of the ilium in the mid-axillary plane. Hip circumference was measured at the level of the greater trochanter of the femur, -around the buttocks through the symphysis pubis.

Obesity and central obesity were assessed by body mass index (BMI), waist-to-hip ratio (WHR) and waist circumference (WC). BMI was calculated as weight (kg) divided by squared height (m^2^) and used to define normal weight (18.5 kg/m^2^ ≤ BMI < 25 kg/m^2^), overweight (25 kg/m^2^ ≤ BMI < 30 kg/m^2^) and obesity (BMI ≥ 30 kg/m^2^) [18,19]. WHR was calculated as waist (cm) divided by hip (cm) circumference. Presence of abdominal obesity was considered in case of elevated WHR (>1.0 for men and >0.85 for women, respectively) or elevated waist circumference (WC) (>102 cm for men and 88 cm for women, respectively) [20-22].

Using the WHO STEPwise approach vigorous physical activity was defined as sustained activity that results in a significant increase in heart and breathing rate for at least ten minutes at a time such as forestry, digging construction, farm work or sporting activity. Moderate intensity physical activity was defined as activity that results in breathing somewhat harder than normal for at least ten minutes such as cleaning, washing, planting and harvesting crops, weaving, woodwork, brisk or quick walking or carrying of light loads. Low intensity physical activity was defined as involving mostly sitting or standing with walking for not more than ten minutes [23].

In addition, information on socioeconomic and demographic data, data on past and current medical history of diabetes and its risk factors (tobacco smoking, alcohol consumption, dietary habits, and physical activity) was obtained from all study participants.

### Data analysis

Means and standard deviations (SD) were used to describe continuous variables. Logistic regression was used to investigate the association of demographic and anthropometric characteristics, blood pressure and lifestyle risk factors to IFG/T2D. Sex, marital status and variables significantly associated (two sided p-value < 0.05) with IFG/T2D in univariate analyses were subsequently included in multivariate logistic regression analyses. Results were reported as Odds Ratios (OR) with corresponding 95% confidence intervals (95% CI). Statistical correlations for the presence of IFG/T2D were assessed between WHR and BMI, BMI and waist circumference, and between sex and markers of central obesity and BMI. Separate models were considered in case of correlation between variables. We carried out direct age standardisation using World Health Organisation New World Population as reference [24]. Regression analysis took into consideration stratification, proportional weights and clustering for participants with main dependent variable having complete data. All statistical analyses were adjusted for the cluster random sampling design by use of the statistical software package STATA/IC (version 11.1).

### Ethical considerations

Prior to study inclusion participants gave informed consent by either signing or thumb printing on the informed consent form, a copy of which was given to the participant. Information obtained from individuals and households and results of the physical and biochemical examinations were kept confidential. All participants received individual feedback on the results of their examinations and were referred where necessary to Biyem-Assi hospital–Yaoundé for appropriate follow up. Data were anonymized for statistical analysis. Ethical clearance for the CAMBoD study was obtained from the Cameroon National Ethics Committee; Yaoundé, Cameroon; ethical clearance for analysis of the 2007 survey was given by the Human Research Ethics Committee (Medical) of the University of the Witwatersrand, Johannesburg, South Africa.

## Results

### Demographic and socio-economic characteristics of study participants

The study sample comprised 1623 participants, 666 men (41.1%) and 957 women (58.9%). Average age was 39.7 (SD 12.9) years at the time of the survey in 2007. Demographic characteristics, i.e. marital status, educational attainment and information on occupation are given in Table 1.

**Table 1:** **Description of study participants according to IFG/T2D status, Yaoundé, Cameroon, 2007**

**Variable**	**All**		**IFG/T2D**		**Non IFG/T2D**		P-value*
	**(n = 1623)**		**(n = 93)**		**(n = 1530)**		
	**N**	**%**	**n**	**%**	**N**	**%**	
**Gender**							
Male	666	41.0	55	45.1	611	40.7	
Female	957	59.0	67	54.9	890	59.3	0.345
**Age group (years)**							
25-24	768	47.3	22	18.0	746	49.7	
35-44	278	17.1	22	18.0	256	17.1	
45-54	337	20.8	38	31.2	299	19.9	
55-64	170	10.5	34	27.9	136	9.1	
65+	70	4.3	6	4.9	64	4.3	<0.001
**Marital status**							
Married	852	52.5	84	68.9	768	51.2	
Single	584	36.0	20	16.4	564	37.6	
Others	187	11.5	18	14.8	169	11.3	<0.001
**Occupation (n = 1526)**							
Civil servant/Private sector	591	38.7	51	46.4	540	38.1	
Self-employed/unpaid subsistence farming	259	16.9	18	16.4	241	17.0	
Student	197	12.9	4	3.6	193	13.6	
Retired/House keeper	288	18.9	30	27.3	258	18.2	
Unemployed	191	12.5	7	6.3	184	12.9	<0.001
**Highest level of education (n = 1607)**							
Never went to school to completed primary school	366	22.8	31	25.6	335	22.5	
Completed secondary school	405	25.2	30	24.8	375	25.2	
Completed high school	391	24.3	27	22.3	364	24.5	
Completed university	445	27.7	33	27.3	412	27.7	0.873
**Weight status**							
Underweight to normal	593	36.5	30	24.6	563	37.5	
Overweight	562	34.6	42	34.4	520	34.6	
Obese	468	28.8	50	41.0	418	27.9	0.003
**Waist-to-hip ratio**							
<0.90(men) or <0.85(women)	1029	63.4	48	39.3	981	65.4	
>0.90(men) or >0.85(women)	594	36.6	74	60.7	520	34.6	<0.001
**Waist circumference**							
≤94(men) ≤ 80 cm(women)	743	45.8	40	32.8	703	46.8	
94 > & < 102(men)80 > &88 cm (women)	313	19.3	18	14.8	295	19.7	
≥102 cm(men) ≥ 88 cm(women)	567	34.9	64	52.4	503	33.5	<0.001
**Hypertension**							
Yes	432	26.6	51	41.8	381	25.4	
No	1191	73.4	71	48.2	1120	74.6	<0.001
**Smoking (n = 1 607)**							
Never	1219	75.9	91	75.8	1128	75.9	
Former	266	16.5	19	15.8	247	16.6	
Current	122	7.6	10	8.3	112	7.5	0.935
**Alcohol consumption**							
Never	269	16.7	19	15.6	250	16.7	
Former	293	18.2	23	18.9	270	18.1	
Current	1051	65.1	80	65.5	971	65.1	0.935
**General physical activity**							
None	628	38.6	37	30.3	591	39.4	
Low intensity	837	51.6	73	59.8	764	50.9	
Moderate activity	87	5.4	4	3.3	83	5.5	
Vigorous activity	71	4.4	8	6.6	63	4.2	0.084
**Walking/cycling for at least 10 minutes (n = 1 609)**							
Yes	1339	83.2	96	80.7	1 243	83.4	
No	270	16.8	23	19.3	247	16.6	0.440
**Activity during recreation/leisure (n = 1614)**							
None	89	5.5	2	1.7	87	5.8	
Low intensity	1394	86.4	105	88.2	1 289	86.2	
Moderate activity	42	2.6	4	3.4	38	2.5	
Vigorous activity	89	5.5	8	6.7	81	5.4	0.251
**Fruit consumption/week (n = 1 471)**							
0/day	299	20.3	26	22.0	273	20.2	
1-2 days	252	17.1	29	24.6	223	16.5	
3-4 days	731	49.7	44	37.3	687	50.8	
5-7 days	189	12.9	19	16.1	170	12.5	0.025
**Vegetable consumption/week(n = 1 555)**							
1-2 days	183	11.8	19	16.1	164	11.4	
3-4 days	407	26.1	37	31.4	370	25.8	
5-7 days	965	62.1	62	52.5	903	62.8	0.074

*Chi-square test for comparison of distribution of variable categories for IFG/T2D versus non IFG/T2D.

### Prevalence of IFG/Type 2 diabetes, hypertension and obesity

Prevalence of T2D was 3.3% (n = 54), 4.2% (n = 28) among men and 2.7% (n = 26) among women. Figures for IFG/T2D were 5.7% (n = 93), respectively 6.8% (n = 45) in men and 5.0% (n = 48) in women. Mean FCG was 5.4 mmol/l. IFG/T2D prevalence increased with age (Table 2). Thirty-one (57.4%) of the 54 respondents considered to have T2D reported diabetes treatment; 42.6% were newly diagnosed cases. Diabetes control was poor: 42.0% of diabetics on treatment had FCG ≥ 6.1 mmol/l, 29.0% had FCG ≥ 7.0 mmol/l.

**Table 2:** **Percentage description of type 2 diabetes (T2D), Impaired fasting glucose (IFG)/T2D, hypertension, waist-hip-ratio, waist circumference and body mass index by age group and by gender among 1623 study participants in Yaoundé, Cameroon, 2007**

		**Men (n = 666)**							**Women (n = 957)**					
		**25-34**	**35-44**	**45-54**	**55-64**	**>65**	**p-value** ^**7**^		**25-34**	**35-44**	**45-54**	**55-64**	**>65**	**p-value** ^**7**^
**N**	**666**	**317**	**100**	**116**	**100**	**33**		**957**	**451**	**178**	**221**	**70**	**37**	
**Variable**	N (%)	N (%)	N (%)	N (%)	N (%)	N (%)		N (%)	N (%)	N (%)	N (%)	N (%)	N (%)	
T2D^1^	28 (4.2)	3 (10.7)	1 (3.6)	8 (28.6)	14 (50.0)	2 (7.1)	<0.001	26 (2.7)	4 (15.4)	4 (15.4)	10 (38.5)	6 (23.1)	2 (7.7)	<0.001
IFG/T2D^2^	45 (6.8)	5 (11.1)	8 (17.8)	9 (20.0)	21 (46.7)	2 (4.4)	<0.001	48 (5.0)	9 (18.8)	8 (16.7)	20 (41.7)	8 (16.6)	3 (6.2)	<0.001
Hypertension^3^	203 (30.5)	37 (18.2)	21 (10.3)	58 (28.6)	59 (29.1)	28 (13.8)	<0.001	229 (23.9)	35 (15.3)	24 (10.5)	101 (44.1)	43 (18.8)	26 (11.4)	<0.001
Weight status^4^														
Normal weight	314 (47.2)	195 (62.1)	38 (12.1)	33 (10.5)	32 (10.2)	16 (5.1)		279 (29.2)	185 (66.3)	38 (13.6)	28 (10.0)	15 (5.4)	13 (4.7)	
Overweight	243 (36.5)	95 (39.1)	41 (16.9)	54 (22.2)	44 (18.1)	9 (3.7)		319 (33.3)	157 (49.2)	60 (18.8)	71 (22.3)	22 (6.9)	9 (2.8)	
Obese	109 (16.4)	27 (24.8)	21 (19.3)	29 (26.6)	24 (22.0)	8 (7.3)	<0.001	359 (37.5)	109 (30.4)	80 (22.3)	122 (33.9)	33 (9.2)	15 (4.2)	<0.001
Waist-hip-ratio^5^														
Normal	414 (62.2)	280 (67.6)	58 (14.0)	40 (9.7)	29 (7.0)	7 (1.7)		615 (64.3)	340 (55.3)	118 (19.2)	116 (18.9)	29 (4.7)	12 (1.9)	
Elevated	252 (37.8)	37 (14.7)	42 (16.7)	76 (30.2)	71 (28.2)	26 (10.3)	<0.001	342 (35.7)	111 (32.5)	60 (17.5)	105 (30.7)	41 (12.0)	25 (7.3)	<0.001
Waist circumference^6^														
Normal	485 (72.8)	287 (59.2)	64 (13.2)	65 (13.4)	52 (10.7)	17 (3.5)		258 (27.0)	185 (71.7)	40 (15.5)	21 (8.1)	8 (3.1)	4 (1.6)	
Increased risk	98 (14.7)	23 (23.5)	17 (17.4)	23 (23.5)	28 (28.6)	7 (7.1)		215 (22.5)	113 (52.6)	33 (15.4)	43 (20.0)	15 (7.0)	11 (5.1)	
Elevated	83 (12.5)	7 (8.4)	19 (22.9)	28 (33.7)	20 (24.1)	9 (10.8)	<0.001	484 (50.6)	153 (31.6)	105 (21.7)	157 (32.4)	47 (9.7)	22 (4.6)	<0.001

^1^FCG > =7.0 mmol/l and /or on diabetes medication.

^2^FCG > =6.1 mmol/l and /or on diabetes medication.

^3^SBP ≥ 140 and /or DBP ≥ 90 mmHg and / or on hypertensive medication.

^4^Normal weight (18.5Kg/m^2^ ≤ BMI < 25Kg/m^2^), overweight (25Kg/m^2^ ≤ BMI < 30Kg/m^2^) and obese (BMI ≥ 30Kg/m^2^ **)**.

^5^Normal waist-hip-ratio < 1.0 for men and <0.85 for women and elevated waist-hip-ratio >1.0 for men and >0.85 for women.

^6^Normal waist circumference < 94 cm for men and <80 cm for women, increased risk (94 cm > WC < 102 cm) for men and (80 cm > WC < 88 cm) for women and elevated ≥102 cm for men and ≥ 88 cm for women.

Prevalence of hypertension was 26.6% and significantly higher in older age groups (Table 2). Of the 432 participants considered to be hypertensive, 93 (21.5%) participants reported receiving treatment and 45 (10.4%) had controlled blood pressure. The majority of study participants were overweight (34.5%) or obese (28.4%). Average BMI of the study population was 27.6 kg/m^2^ (SD 5.4)) and was lower in men (25.9 (SD4.1) kg/m^2^ than women (28.7 (SD 5.8) kg/m^2)^. Women were more often obese (37.0%) than men (16.1%); obesity was most prevalent in the age group 45–54 years. More than a third of the study participants (36.6%) showed elevated WHR, slightly more pronounced in men (37.8%) than women (35.7%). Elevated waist circumference was present in 34.9% of the study participants and was four times as high in women (50.6%) as in men (12.5%). For both parameters of central obesity a significant relation with age was observed.

### Prevalence of lifestyle factors – tobacco consumption, alcohol consumption, physical activity and dietary habits

Most survey respondents were non-smokers (75.3%), 7.7% reported to be current smokers, the majority of them being men (89.1%). Prevalence of current smoking was most pronounced in the age group 35–44 years, older age groups tended to report less current smoking. On average current smokers started smoking at 21.2 (SD 5.9) years. Less than a third (28.%) of those who reported to have smoked previously indicated to have stopped because of health related problems; on average they reported to have quit smoking at age 34.7 (SD 11.1) years.

Alcohol consumption was common with 65.5% of participants reporting current alcoholic beverage consumption such as beer, wine, spirit or local brews as palm wine, corn beer, “bilibili”, “arki” or “afofo” and was most prominent in those aged 25–34 years (men 91.2%, women 78.5%). Men more often reported current alcohol consumption than women and were less often abstainers. Beer was the most consumed alcoholic drink, specifically in men (70.9%, women 49.8%) followed by consumption of wine, palm wine, whisky, “bilibili” or corn beer and “arki”.

Most respondents (61.5%) reported physical activity, mostly low intensity physical activity (51.8%). Vigorous physical activity was only reported by 4.3% of the participants. In general, intense physical activities were reported more often by younger age groups and more often by men than women (data not shown). For leisure/recreational activities most study participants (85.9%) reported light activities, vigorous intensity was only reported by 6.1% of the study participants.

On average fruit consumption was reported on 3 days/week, vegetable consumption on 2.4 days/week; less than 2.5% of respondents reported any vegetable consumption, whereas 13.4% did not report any fruit consumption. Older women reported highest fruit consumption.

### Risk factors for IFG/T2D

Diabetic participants were often older, obese, hypertensive, had elevated waist circumference and WHR, and were less often single and more often retired (Table 1). Age was significantly associated with IFG/T2D (Table 3). Being obese was associated with increased likelihood of IFG/T2D when compared to normal weight participants (OR: 2.24, 95% CI: 1.33–3.78). Both markers of abdominal obesity were associated with IFG/T2D, OR for elevated WHR was 2.91 (95% CI: 1.99–4.25), OR for high waist circumference was 2.23 (95% CI: 1.45–3.46). Hypertension (OR = 2.11, 95% CI: 1.42–3.13) was also associated with IGF/T2D (Table 3).

**Table 3:** **Risk factors for IFG/T2D in study participants aged 25 years and older in Yaoundé, Cameroon, 2007**

**Factors**	**Univariate logistic regression**				**Multivariate logistic regression**			
	**OR**	**95% CI**	**P**	**p for trend**	**OR**	**95% CI**	**p**	**p for trend**
**Gender**								
Female	1				1			
Male	1.19	0.82-1.74	0.352	0.352	1.23	0.73-2.07	0.443	0.332
**Age Group**								
25-34	1				1			
35-44	2.91	1.59-5.31	0.000		2.65	1.27-5.53	0.009	
45-54	4.31	2.53-7.32	0.000		3.04	1.50-6.14	0.002	
55-64	8.48	4.67-15.37	0.000		5.69	2.52-12.83	0.000	
65+	3.18	1.28-7.90	0.013	<0.001	1.88	0.60-5.86	0.275	<0.001
**Marital status**								
Married	1							
Single	0.32	0.19-0.54	0.000					
Others	0.97	0.50-1.88	0.937	0.119				
**Weight status**								
Normal	1				1			
Overweight	1.52	0.90-2.55	0.117		0.92	0.47-1.80	0.810	
Obese	2.24	1.33-3.78	0.002	0.002	1.14	0.56-2.34	0.704	0.503
**Waist-hip ratio**								
<1.0 (men) or <0.85 (women)	1				1			
>1.0 (men) or >0.85 (women)	2.91	1.99-4.25	0.000	<0.001	2.21	1.22-4.01	0.009	0.008
**Waist circumference in cm**								
≤94 (men) or ≤80 (women)	1				1			
94 > & < 102 (men) or 80 > & < 88 (women)	1.07	0.59-1.96	0.821		0.71	0.33-1.53		
≥102 (men) or ≥88 (women)	2.23	1.45-3.46	0.000	<0.001	1.08	0.48-2.47	0.850	0.798
**Prevalence of hypertension**								
No	1				1			
Yes	2.11	1.42-3.13	0.000	<0.001	1.58	0.92-2.72	0.100	0.145
**Smoking**								
Never	1							
Former	0.95	0.57-1.60	0.857					
Current	1.11	0.56-2.19	0.771	0.888				
**Alcohol consumption**								
Never drank	1							
Former	1.12	0.57-2.21	0.743					
Current	1.08	0.65-1.81	0.758	0.820				
**General physical activity**								
None	1							
Low intensity	1.53	0.96-2.42	0.073					
Moderate activity	0.77	0.26-2.29	0.637					
Vigorous activity	2.03	0.88-4.69	0.098	0.111				

Gender and all variables significantly associated with IFG/T2D in univariate models were included in multivariate logistic regression models to further analyse the association with IFG/T2D. The two markers of abdominal obesity were considered in separate multivariate logistic regression analysis due to their high correlation. Both markers were highly associated with IFG/T2D in multivariate adjusted models. Hosmer–Lemeshow goodness-of-fit test confirmed the appropriateness of the two multivariate adjusted models.

The factors that maintained their statistical significance after multivariate adjustment were age (35–44 years: OR = 2.63, 95% CI: 1.26–5.51, p = 0.010; 45–54 years: OR = 3.05, 95% CI: 1.50–6.22, p < 0.002 and 55–64 years: OR = 5.88, 95% CI: 2.59–13.37, p < 0.001) and elevated WHR (OR = 2.21, 95% CI: 1.22–4.01, p = 0.009) (Table 3).

## Discussion and conclusions

The current study assessed the prevalence of and the risk factor profile for IFG/T2D in the general adult population in Biyem-Assi, Yaoundé, Cameroon and observed age and central obesity to be the most important risk factors associated with prevalence of IFG/T2D.

An increase in the prevalence of diabetes in this urban area was observed from 2.0% in 1999 [4] to 3.3% in 2007. Regional and comparative prevalence in Africa was 4.9% and 5.7% respectively and ranged from 4.4% to 7.0% in 2013 depending on the country’s economic development [25]. A higher urban prevalence for diabetes was observed in Tanzania for Asian Indians in 1991 (9.1%) and South Africa for Native Africans in 1993 (8.0%) [4].

In Zambia, the combined prevalence of impaired glucose levels or diabetes in 2011 was 4.0% (impaired glucose levels or diabetes defined as fasting glucose ≥ 5.51 mmol/l) [26], higher than for this study most likely due to a lower cut off point for IFG levels. A similar study in China using the same cut off points for IFG and T2D as in our study showed a higher prevalence of diagnosed diabetes, undiagnosed diabetes and IFG in adults respectively; 1.3%, 4.2% and 7.3% [27], possibly in part due to increased levels of physical inactivity, high levels of alcohol consumption and poor dietary habits. Beneficial effects of lifestyle behaviour to prevent or delay the onset of diabetes and other health related benefits have been demonstrated [28-30]. Prevalence estimates observed in the current study were lower than estimates for diabetes mellitus (5.7%) and IFG (4.5%) reported by a systematic review and meta-analysis in sub-Saharan Africa published in 2013 [31]. Disparities in prevalence are probably in part due to differences in health determinants across sub-Saharan Africa [31]. IFG is a risk state prior to diabetes and ideally diabetes prevention strategies identify and focus on individuals at pre-diabetes state. Newly diagnosed cases represented 42.6% in our study in contrast to more than 70% of newly diagnosed cases reported for surveys [4]. This difference can be most likely attributed to on-going sensitisation campaigns in the study area to curb diabetes [12]. The observed associations of age and central obesity with IFG/T2D in this Cameroonian population are in accordance with previous international observations [4-6], in particular central obesity was observed to be a good predictor of IFG/T2D. Waist circumference has previously been shown in a Cameroonian population to be positively associated to all obesity related abnormalities [32].

We observed no association between fruit and vegetable consumption and IFG/T2D, despite previous reports on an inverse association of these low energy density, low glycaemic load, and high fibre and micronutrient content foods with risk of developing IFG/T2D [33,34]. Our results may in part be explained by poor quantification of the type and amounts of fruits and vegetables consumed with the interviewer-administered questionnaire and with seasonal availability of these produce on the local market.

Likewise, we observed no associations for current and former smokers with IFG/T2D, nor an inverse association for moderate alcohol intake or physical activity with IFG/T2D [35-38] pointing towards methodological challenges of the WHO-STEPS tool applied for assessment of risk factors in this population in Cameroon. These may entail for example for physical activity, difficulties in interpretation of the physical activity questions, e.g. perception of categories of physical activity on the one side and on the other side the notion that some leisure/recreational physical activities in Cameroon are rather considered to be social occasions coupled with entertainment including alcohol and food consumption potentially offsetting the beneficial effects that accrue from physical activity.

The results reported in the current study are based on the CAMBoD survey conducted in the adult population of Biyem-Assi, Yaoundé, Cameroon in 2007. In comparison with a survey conducted in the area in 2003 increased prevalence for IFG/T2D ((3.7% (2003) to 4.1% (2007), hypertension (17.1% (2003) to 20.4% (2007)) and obesity (14.6% (2003) to 18.3% (2007); all age-standardized data) were noted. Our study showed that T2D, IFG/T2D, hypertension and adiposity affects particularly people in the age group 45-54 years. Prevalence has increased though less than for any of the top 10 countries in the African region for which increasing prevalence of 6.3% to 15.4% was reported in 2013 [25]. Urban settlements are fast becoming diabetic and obesity epi-centres and increasingly people at younger ages are observed to develop diabetes [2,4,5]. The high burden of diabetes in low and middle income countries especially in sub-Saharan Africa [25,31] calls for increased efforts to address individual lifestyle changes to reduce modifiable IFG/T2D risk factors [28,29], particularly central obesity, to counteract accelerating prevalence rates of IFG/T2D in a population undergoing rapid urbanisation and epidemiologic transition with limited health resources accentuated by negligible health expenditure on diabetes.

### Limitations and advantages of the study

The current cross-sectional study was conducted in an urban setting in Yaoundé and may thus not be generalizable to the rural population in Cameroon. Missing information in the survey population may further limit the generalizability of the study results. For definition of pre-diabetes, we used IFG instead of IGT, which considering that FCG may potentially fail to identify previously undiagnosed diabetes, could have introduced misclassification of IFG/T2D. Participants on anti-diabetic medication were considered to be diabetic, however, some may have been prescribed anti-diabetic medication at a pre-diabetes stage, which might have resulted in some misclassification of IFG and T2D.

Despite these limitations and taking into consideration feasibility, reproducibility, acceptability and local context our study provided prevalence data of T2D, IFG/T2D, hypertension and adiposity by sex and age group strata for an urban Cameroonian population. The study seeks to enhance public awareness on the seriousness of diabetes and its complications and the need for prevention strategies especially in people less than 45 years of age.
